# Triacontanol Boosts Soybean Nodulation via *GmHSP26*-Mediated Antioxidant Enhancement

**DOI:** 10.3390/plants15101572

**Published:** 2026-05-21

**Authors:** Bingjie Niu, Minglei Cheng, Xudong Lu, Lili Sun, Shuang Lu, Jinke Guo, Hongyan Zhu, Lixiang Wang

**Affiliations:** 1 Shanxi Houji Laboratory, College of Agriculture, Shanxi Agricultural University, Taigu 030801, China; 242005@sxau.edu.cn (B.N.); 20235026@stu.sxau.edu.cn (M.C.); 20232128@stu.sxau.edu.cn (X.L.); b20221024@stu.sxau.edu.cn (L.S.); 202430187@stu.sxau.edu.cn (S.L.); 202520124@stu.sxau.edu.cn (J.G.); 2 College of Resources and Environment, Shanxi Agricultural University, Taigu 030801, China; 3 Department of Plant and Soil Sciences, University of Kentucky, Lexington, KY 40506, USA

**Keywords:** soybean, triacontanol (TRIA), nodulation, transcriptome, antioxidant

## Abstract

Soybean (*Glycine max* (L.) Merr.) is a globally crucial food crop and a model plant for studying symbiotic nitrogen fixation in legumes. Triacontanol (TRIA) is a natural plant growth regulator that enhances photosynthetic efficiency, stress tolerance, antioxidant enzyme activities and yield in crops. However, its regulatory role in nodulation and nitrogen fixation in legumes remains unclear. In this study, soybean seedlings inoculated with *Bradyrhizobium japonicum* strain USDA110 were treated with different concentrations of TRIA (0, 0.33, 0.5, 1 and 2 μg/mL). Then, oxidative stress indicators and comparative transcriptomic analysis were performed to check the oxidative status and screen the candidate genes under TRIA treatment. Our results showed that the 0.5 μg/mL TRIA treatment produced the greatest nodule number. TRIA treatment significantly induced antioxidant responses in soybean roots. Comparative transcriptome identified 867 differentially expressed genes (DEGs), Gene Ontology and Kyoto Encyclopedia of Genes and Genomes enrichment analyses of DEGs revealed that a large number of genes were enriched in pathways related to oxidative activity. Combined with the expression pattern, we identified a Glutathione S-Transferase family gene, *GmHSP26* (*Glyma.07G139700*), whose expression was induced by both TRIA and rhizobial infection, with its promoter activity was activated throughout the entire process of nodule development. Further function study using overexpression and gene editing proved that *GmHSP26* was a positive regulator of soybean nodulation. Collectively, this study identifies the optimal TRIA concentration for promoting soybean nodulation, reveals the function and mechanism of *GmHSP26* in response to TRIA-regulated nodulation, and provides a theoretical basis and genetic resource for enhancing nodulation and nitrogen fixation in leguminous crops through exogenous growth regulators.

## 1. Introduction

Biological nitrogen fixation is a key process in nature that converts atmospheric dinitrogen into biologically available nitrogen [1]. Among the various nitrogen-fixing systems, the symbiotic nitrogen fixation (SNF) system between legumes and rhizobia has attracted considerable attention due to its exceptionally high efficiency [2,3]. SNF is a biological process characterized by “on-demand production and fine-tuned regulation” [4]. The host plant supplies energy through photosynthetic products (e.g., sucrose) and maintains a microaerophilic environment via leghemoglobin, while the autoregulation of nodulation (AON) mechanism prevents excessive energy consumption [5]. This bidirectional mutualistic metabolic integration is far superior to the passive assimilation mode that relies on exogenous nitrogen fertilizers [6]. SNF can meet 50–80% of the nitrogen demand of leguminous plants, thereby reducing the application of chemical fertilizers and lowering agricultural production costs [7]. Furthermore, a healthy symbiotic system helps maintain soil microbial diversity and promotes carbon–nitrogen balance, serving as a core foundation for achieving green and low-carbon agriculture as well as sustainable development [8].

The process of nodulation and nitrogen fixation is not determined solely by rhizobia, but is synergistically influenced by multiple factors including the physiological status of the host plant, environmental conditions, and exogenous regulatory substances [9,10,11]. Among these, plant growth regulators (PGRs) have gradually become important tools for regulating nodulation due to their wide range of sources, high efficacy at low concentrations, and broad applicability [12]. Unlike microbial inoculants, which are limited by geographic, soil, and crop specificity [13], PGRs (e.g., brassinosteroids, gibberellins, abscisic acid, and humic acid) have demonstrated significant growth-promoting and regulatory effects in various crops [14,15]. They can directly or indirectly affect nodule formation and function by improving the overall physiological status of plants and modulating carbon–nitrogen metabolism and antioxidant systems.

As a natural long-chain alcohol plant growth regulator, triacontanol (TRIA) can be extracted from raw materials such as beeswax and plant roots [16,17]. It is characterized by broad crop adaptability and significant, persistent effects. Previous studies have shown that TRIA increases leaf chlorophyll content and photosynthetic efficiency, enhances antioxidant enzyme activities, alleviates damage caused by heavy metals, drought, high temperature, and other stresses, and significantly improves the yield and quality of crops such as soybean, peanut, and maize [18,19,20,21]. Regarding SNF, preliminary evidence indicates that TRIA treatment improves nitrogenase activity, photosynthesis, and biological yield in hyacinth bean (*Lablab purpureus* (L.) Sweet), suggesting that it may influence rhizobial infection and nodule development by providing sufficient energy substrates and modulating root redox balance [22]. However, systematic research on the effects of TRIA on nodulation and nitrogen fixation in legumes remains scarce, and in particular, whether and how it affects nodule number, nodule development processes, and the associated molecular mechanisms has yet to be thoroughly explored.

Therefore, systematically investigating the regulatory effects of TRIA on nodulation and nitrogen fixation in legumes, and elucidating its optimal application concentration, physiological mechanisms, and key gene pathways, will not only expand the application boundaries of plant growth regulators in green agriculture but also provide new insights for synergistically enhancing SNF efficiency and reducing dependence on chemical nitrogen fertilizers through exogenous regulation. This research holds significant theoretical and practical value for promoting the construction of a “plant–microbe–plant growth regulator” synergistic strategy in the development of green agriculture.

## 2. Results

### 2.1. Effect of TRIA Treatment on Soybean Nodulation

**Figure 1:**
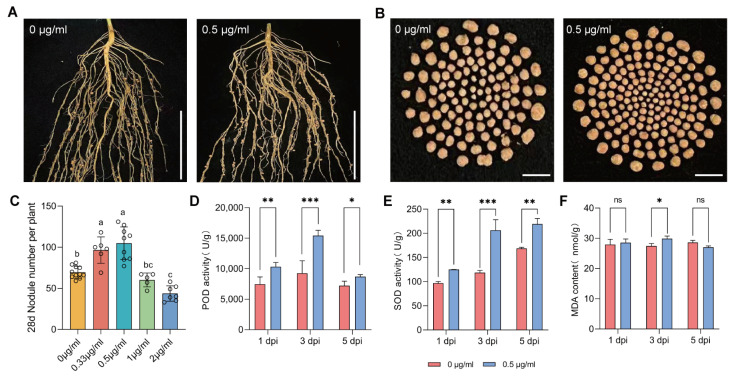
Soybean nodule phenotype at 28 dpi and oxidative activity at 1, 3, and 5 dpi under TRIA treatment. (**A**) Comparison of soybean root nodulation phenotypes under 0 μg/mL and 0.5 μg/mL TRIA treatments. Scale bar = 10 cm. (**B**) Comparison of nodule numbers in soybean roots under 0 μg/mL and 0.5 μg/mL TRIA treatments. Scale bar = 1 cm. (**C**) Statistics of nodule numbers in soybean under gradient concentrations of TRIA (n = 5–10). Tukey HSD multiple comparisons (α = 0.05) results are marked with letters; different lowercase letters indicate significant differences between treatments (*p* ≤ 0.05), while the same letters indicate no significant difference. (**D**) POD activity under different treatments and time points (n = 3). (**E**) SOD activity under different treatments and time points (n = 3). (**F**) MDA content under different treatments and time points (n = 3). (**D**–**F**) Data are presented as mean ±standard error of the mean (SEM). Asterisks indicate significant differences based on two-tailed Student’s *t*-test: * represents *p* ≤ 0.05, ** represents *p* ≤ 0.01, *** represents *p* ≤ 0.001. ns represents no significant difference (*p* > 0.05).

To investigate the effect of TRIA treatment on soybean nodulation, we diluted a 0.1% triacontanol microemulsion with water to prepare a series of concentrations of 0.33 μg/mL, 0.5 μg/mL, 1 μg/mL, and 2 μg/mL, with water (0 μg/mL) serving as the control (CK). Soybean seedlings with fully expanded true leaves were inoculated with rhizobia strain USDA110, after which the roots were treated with different concentrations of TRIA. Root nodulation was observed at 14 and 28 dpi (days post inoculation). The results showed that as the TRIA concentration increased, the nodule number per root system at 28 dpi first increased and then decreased, with the highest nodule number observed under the 0.5 μg/mL TRIA treatment, which was significantly higher than CK (Figure 1A–C). The highest nodule number was also observed at 14 dpi under the 0.5 μg/mL TRIA treatment (Figure S1).

**Figure 2:**
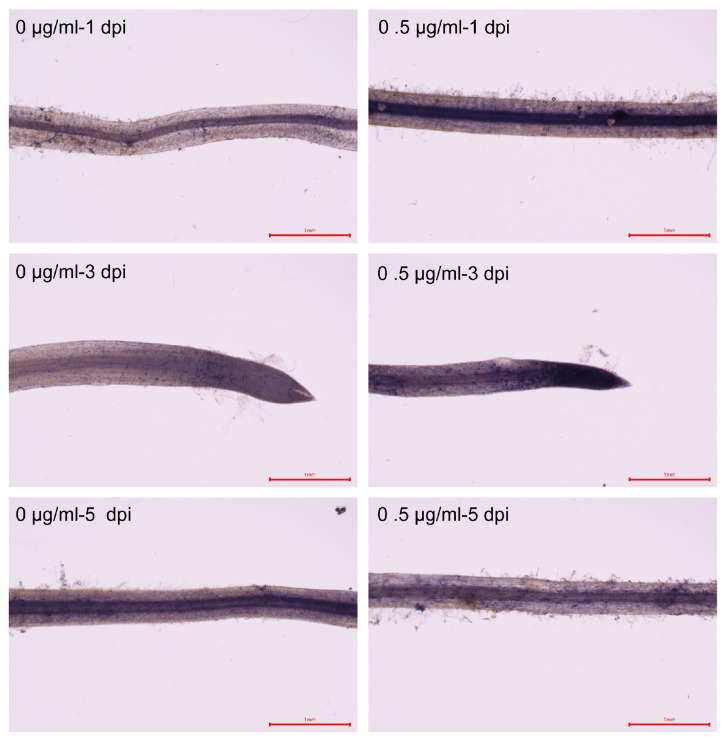
Nitroblue Tetrazolium (NBT) staining of roots treated with 0 μg/mL and 0.5 μg/mL TRIA for 1, 3, and 5 dpi (n = 3). Magnification: 3×. Scale bar = 1 mm.

To investigate whether it regulates nodulation by affecting the oxidative status of the host, we further examined the oxidative status of soybean roots treated with 0.5 μg/mL TRIA at 1, 3, and 5 dpi. The results showed that the activities of SOD (Superoxide Dismutase) and POD (Peroxidase) in the roots were significantly higher than those of the control at all three time points (Figure 1D,E), whereas the MDA (Malondialdehyde) content was significantly higher than that of the control only at 3 dpi, increased by approximately 8.7% (Figure 1F). To visually reflect the level of oxidative stress, Nitroblue Tetrazolium (NBT) staining was used to detect the accumulation of superoxide anions in the root system. NBT staining of the roots revealed that, over time, the staining intensity of the control group gradually increased. In the treatment group, the staining was deepest at 3 dpi and became lighter by 5 dpi, and the changes in staining intensity in the treatment group were more pronounced and rapid (Figure 2).

### 2.2. Transcriptome Analysis of TRIA-Induced Regulation of Root Gene Expression

To identify the genes that response to TRIA treatment under rhizobial inoculation, we performed comparative transcriptome analysis using soybean roots from the CK and 0.5 μg/mL TRIA treatment at 1, 3, and 5 dpi. The transcriptome sequencing yielded a total of 18 samples from six groups (each group with three biological replicates). Transcriptome sequencing was performed on these samples. After filtering the raw data, a total of 114.25 Gb of clean data was obtained. For all 18 biological samples, the Q20 base percentage exceeded 98%, the Q30 base percentage exceeded 95%, and the GC content ranged from 43.36% to 43.62% (Table S1). Principal component analysis and intergroup correlation analysis revealed high intragroup aggregation and clear intergroup separation among the six groups, indicating good transcriptome data quality and significant differences between groups, making the data suitable for further analysis (Figure S2A,B).

**Figure 3:**
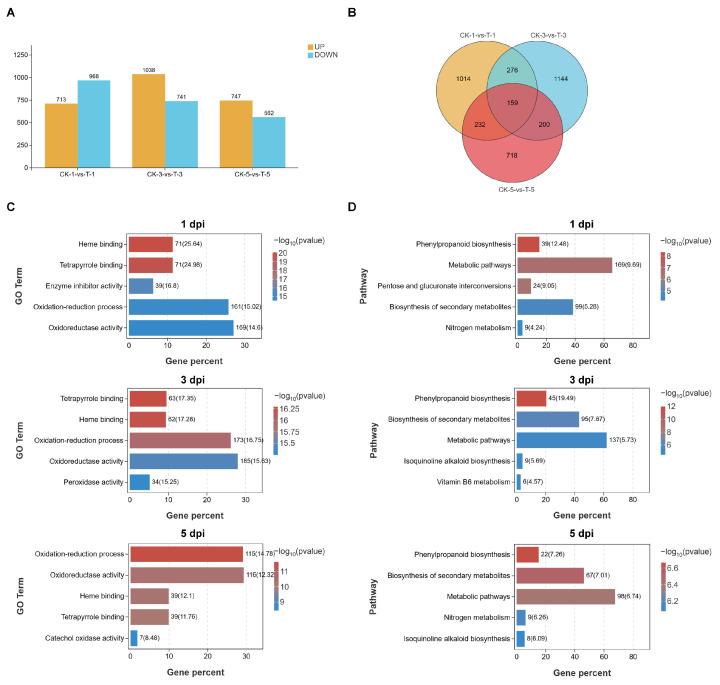
Analysis of DEGs under TRIA treatment. (**A**) Bar chart showing the number of DEGs at different treatment time points. (**B**) Venn diagram of DEGs at different treatment time points. (**C**) GO enrichment analysis of DEGs at different treatment time points. From (**top**) to (**bottom**): GO enrichment analysis of DEGs at 1, 3, and 5 dpi, respectively. Yellow represents CK-1 vs. T-1, blue represents CK-3 vs. T-3, red represents CK-5 vs. T-5, and the overlapping area represents shared DEGs. (**D**) KEGG enrichment analysis of DEGs at different treatment time points. From (**top**) to (**bottom**): KEGG enrichment analysis of DEGs at 1, 3, and 5 dpi, respectively. Colors indicate the significance of differences: red represents higher significance; blue represents lower significance.

Differentially expressed genes (DEGs) were screened using the criteria of a *p* < 0.05 and |fold change| > 2. As shown in Figure 3A, a total of 1699 DEGs were detected between the TRIA and control treatment at 1 dpi, of which 731 genes were upregulated and 968 genes were downregulated. At 3 dpi, a total of 1779 DEGs were detected, including 1038 upregulated and 741 downregulated genes. At 5 dpi, a total of 1309 DEGs were detected, comprising 747 upregulated and 562 downregulated genes. The Venn diagram revealed that 159 genes were differentially expressed at all three time points. A total of 867 DEGs were identified in the intersections of at least two time points, including those common to all three time points (Figure 3B).

Gene Ontology (GO) enrichment analysis was performed on the DEGs between the TRIA treatment and CK at the three time points. As shown in Figure 3C, at 1 dpi and 3 dpi, the GO terms were significantly enriched in “Heme binding” and “Tetrapyrrole binding”; at 5 dpi, the GO terms were significantly enriched in “Oxidation-reduction process” and “Oxidoreductase activity”. GO enrichment analysis was performed separately for the upregulated and downregulated DEGs at 1, 3, and 5 dpi. The results showed that in the upregulated DEGs, terms related to oxidoreductase activity were increasingly enriched with prolonged treatment time, whereas the trend was opposite for the downregulated DEGs (Figure S3). Kyoto Encyclopedia of Genes and Genomes (KEGG) enrichment analysis across the three time points revealed that at any given time point, the DEGs were significantly enriched in the pathways of “Phenylpropanoid biosynthesis”, “Biosynthesis of secondary metabolites”, and “Metabolic pathways” (Figure 3D). Further KEGG enrichment analysis was performed on the upregulated and downregulated DEGs at 1, 3, and 5 dpi, respectively. The results showed that the upregulated and downregulated DEGs at each time point accounted for a large proportion in metabolism-related pathways (Figure S4).

For a subset of DEGs with relatively clear expression trends, qPCR was performed to verify whether their expression trends were consistent with the transcriptome data. As shown in Figure S5, the expression levels of *Glyma.07G139700*, *Glyma.01G028500*, *Glyma.06G170300*, *Glyma.08G341400*, *Glyma.06G286600*, and *Glyma.11G198500* were upregulated, while those of *Glyma.09G034700* and *Glyma.10G151000* were downregulated, as determined by both qPCR and transcriptome data, thereby further demonstrating the reliability of the transcriptome data.

### 2.3. Analysis of TRIA-Induced Nodulation-Related Gene Expression

**Figure 4:**
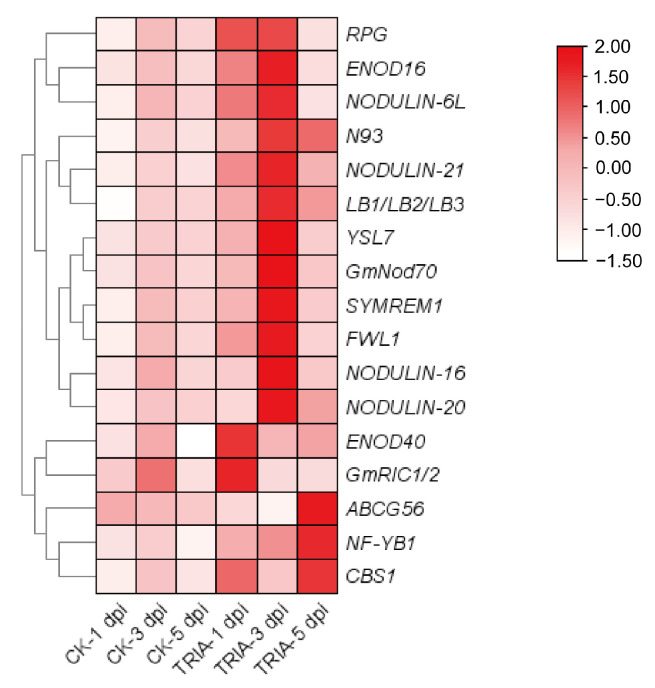
Expression analysis of genes associated with nodulation in transcriptome data. The heatmap was generated using FPKM values with row-wise Z-score normalization. The color scale represents Z-scored expression levels, where red indicates higher expression relative to the gene’s mean across all samples, white indicates lower expression.

To further check the expression status of the nodulation-related genes under TRIA treatment, we analyzed the reported nodulation genes among the 867 DEGs [23]. The results showed that at 1 dpi, *ENOD40* (early nodulin 40) and *GmRIC1/2* (CLE-related protein RIC1/2) exhibited the highest expression levels; at 3 dpi, *RPG* (Rhizobium-directed polar growth), *ENOD16* (early nodulin 16), *NODULIN-6L* (early nodulin-like protein 6), *N93* (Early nodulin 93), *NODULIN-21* (early nodulin-like protein 21), *LB1* (Leghemoglobin 1), *LB2* (Leghemoglobin 2) and *LB3* (Leghemoglobin 3), *YSL7* (YELLOW STRIPE-LIKE 7), *Nod70* (early nodulin 70), *SYMREM1* (Symbiotic Remorin 1), *FWL1* (FW2.2-LIKE 1), *NODULIN-16* (early nodulin 16), and *NODULIN-20* (early nodulin 20) exhibited the highest expression levels expression; and at 5 dpi, *ABCG56* (ATP-binding cassette subfamily G member 56), N*F-YB1* (Nuclear Factor Y, Subunit B1), and *CBS1* (Cystathionine Beta-Synthase) exhibited the highest expression levels (Figure 4). These expression changes in the nodulation-related genes further indicate that TRIA treatment can affect soybean nodulation.

### 2.4. WGCNA Reveals TRIA-Induced Yellow 4 Module and the Hub Gene GmHSP26

**Figure 5:**
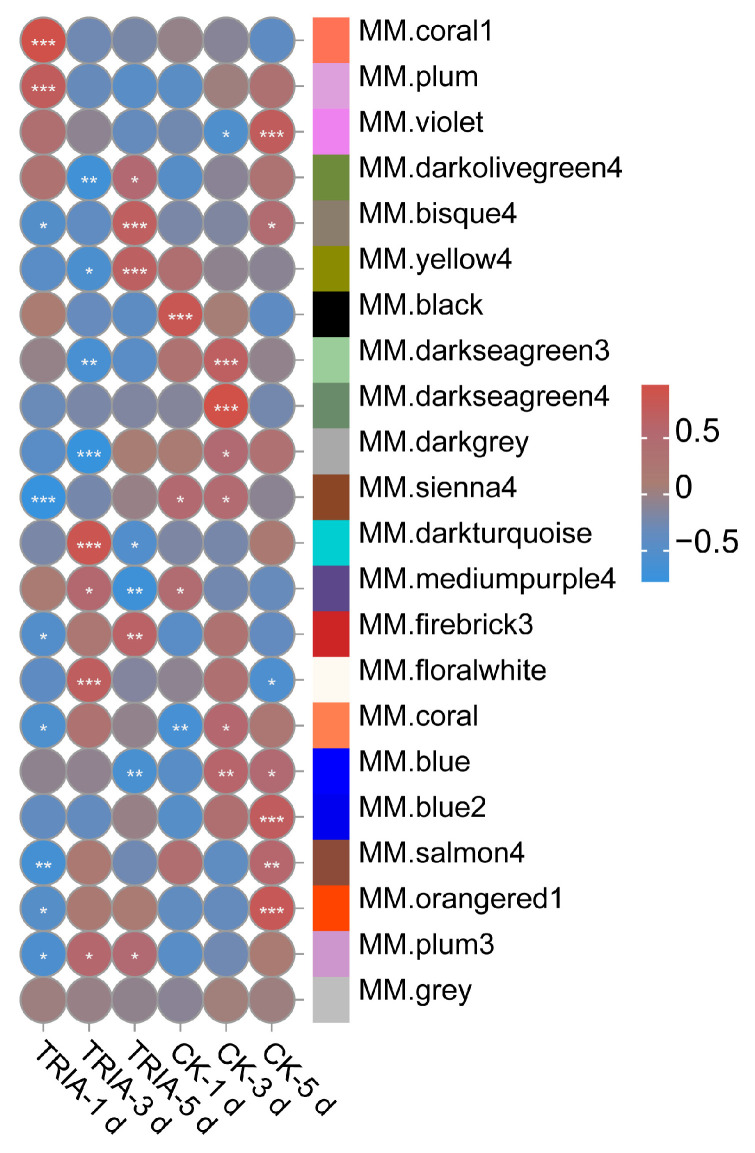
Module-treatment correlation matrix. Pearson correlation analysis: * represents *p* ≤ 0.05, ** represents *p* ≤ 0.01, *** represents *p* ≤ 0.001.

To identify candidate genes that respond to TRIA-influenced nodule changes, we performed weighted gene co-expression network analysis (WGCNA) on all detected genes, resulting in 22 expression modules (Figure S6). By comparing the expression patterns between the two treatments, we focused on the yellow 4 module. In this module, under CK treatment, the expression levels at the three time points showed no significant change; in contrast, under TRIA treatment, gene expression in this module initially exhibited a significant decreasing trend over time and then increased markedly at 5 dpi (Figure 5 and Figure S7), indicating strong induction by TRIA. The yellow 4 module contained a total of 316 genes (Table 1). Among them, 41 genes had module membership (MM) > 0.9. After removing genes without significant differential expression, we identified the hub gene with the highest connectivity, *Glyma.07G139700* (*GmHSP26*) (Table S2).

### 2.5. Expression Patterns and Subcellular Localization of GmHSP26 in Soybean

**Figure 6:**
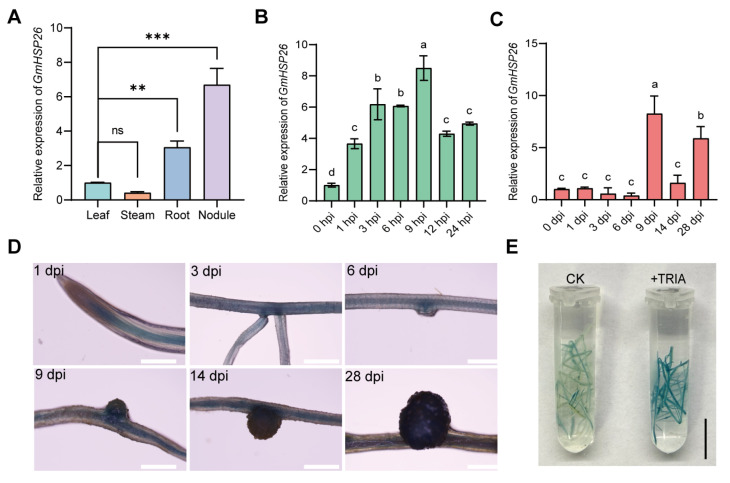
Characteristics of *GmHSP26*. (**A**) Tissue-specific expression levels of *GmHSP26* in different soybean tissues at 14 dpi (n = 3). Using the leaf group as the control, Dunnett’s test was used for multiple comparisons. Asterisks indicate the significance levels compared with the leaf group: ** represents *p* ≤ 0.01, *** represents *p* ≤ 0.001. ns represents no significant difference (*p* > 0.05). (**B**) Expression levels of *GmHSP26* in soybean roots at short time points after inoculation (n = 3). (**C**) Expression levels of *GmHSP26* in soybean roots at long time points after inoculation (n = 3). (**B**,**C**) Tukey HSD multiple comparisons (α = 0.05) results are marked with letters; different lowercase letters indicate significant differences between treatments (*p* ≤ 0.05), while the same letters indicate no significant difference. (**D**) Staining analysis of *proGmHSP26-GUS* at 1, 3, 6, 9, 14, and 28 dpi (n = 3). Magnification: 3×. Scale bar = 1 mm. (**E**) *ProGmHSP26-GUS* staining analysis of CK and TRIA treatment (n = 3). Scale bar = 1 cm.

To detect the response of *GmHSP26* expression to rhizobium inoculation, we first examined the expression level of *GmHSP26* in various soybean tissues and found that its expression in roots and nodules was significantly higher than that in leaves and stems (Figure 6A). To determine whether *GmHSP26* expression is induced by rhizobia, we measured its expression levels at different time points. The results showed that at early time points, *GmHSP26* responded to rhizobial induction at multiple time points. Overall, with increasing time post-inoculation, the expression level first increased and then decreased, peaking at 9 hpi (hours post inoculation) (Figure 6B). At later time points, the expression level of *GmHSP26* initially decreased and then increased with prolonged inoculation time. From 1 to 6 dpi, the expression level was not significantly different from the CK, but it increased significantly at 9 and 28 dpi (Figure 6C). These results indicate that *GmHSP26* expression exhibits fluctuating changes in response to rhizobial induction.

We further applied histochemical staining to check the tissue-specific expression pattern of *proGmHSP26-GUS* during soybean nodulation. Our results showed that at 3 dpi, *GmHSP26* was specifically expressed in the root cap and pericycle. At 6 dpi, expression was detected in lateral root primordia and nodule primordia. At 9 and 14 dpi, expression was observed in young nodules and mature nodules (Figure 6D). *ProHSP26*-GUS staining indicates that TRIA treatment can significantly enhance *GmHSP26* promoter activity (Figure 6E). Confocal microscopy analysis showed that GmHSP26-GFP was predominantly localized to the nucleus and cytoplasm, as evidenced by its extensive colocalization with the 35S-NLS-mCherry (nucleus marker) and 35S-Cyt-mCherry (cytoplasm marker) (Figure S8).

### 2.6. Functional Characterization of GmHSP26 in Soybean Nodulation via Overexpression and Gene Editing

**Figure 7:**
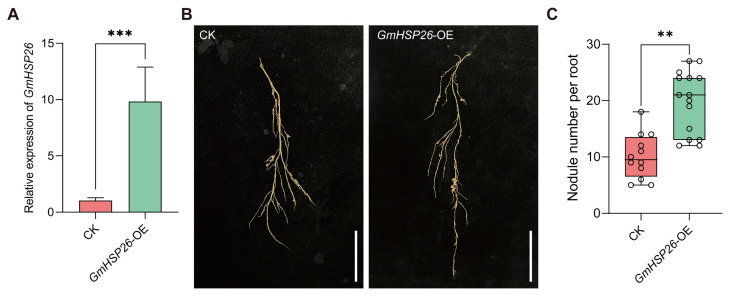
Functional analysis of *GmHSP26*. (**A**) Detection of *GmHSP26* expression levels in CK and *GmHSP26*-OE. (**B**) Comparison of transformed hairy roots between CK and *GmHSP26*-OE. Scale bar = 5 cm. (**C**) Comparison of nodule numbers between CK and *GmHSP26*-OE (n = 12–15 hairy roots, 14 dpi). (**A**,**C**) Data are presented as mean ± standard error of the mean (SEM). Asterisks indicate significant differences based on two-tailed Student’s *t*-test. ** represents *p* ≤ 0.01; *** represents *p* ≤ 0.001.

To determine the function of *GmHSP26* in soybean nodulation, we first used the hairy root transformation method to introduce the *GmHSP26* overexpression vector (*GmHSP26*-OE) into soybean, with the empty vector (CK) as a control, and observed the nodulation status of positive transformed roots at 14 dpi. QRT-PCR verification showed that the expression level of *GmHSP26* in *GmHSP26*-OE hairy roots was significantly higher than that in the control, reaching 9 times that of CK (Figure 7A), confirming successful overexpression of the gene and its suitability for subsequent phenotypic analysis. Analysis of the nodulation phenotype of positive roots in the *GmHSP26*-OE roots revealed that the average nodule number per hairy root was 19.8, compared to 10.08 in the control, indicating that the nodule number in *GmHSP26*-OE was significantly higher than that in CK (Figure 7B,C).

**Figure 8:**
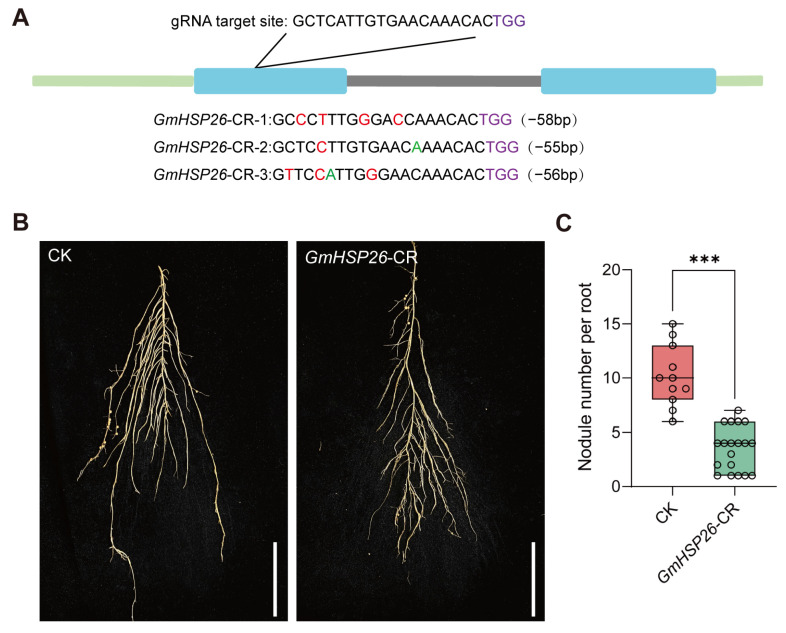
*GmHSP26*-CR suppresses root nodule formation. (**A**) Identification of *GmHSP26* knockout alleles in hairy roots generated by CRISPR/Cas9. In the figure, the light green regions represent untranslated region regions, the blue regions represent exon regions, and the gray regions represent intron regions. The red letters indicate where the base has undergone mutation, and the green letters indicate where the base has been added. (**B**) Comparison of transformed hairy roots between CK and *GmHSP26*-CR. Scale bar = 5 cm. (**C**) Comparison of nodule numbers of *GmHSP26* in CK and *GmHSP26*-CR (n = 11–19 hairy roots, 14 dpi). Data are presented as mean ± standard error of the mean (SEM). Asterisks indicate significant differences based on two-tailed Student’s *t*-test. *** represents *p* ≤ 0.001.

Additionally, we performed gene knockout of *GmHSP26* using gene editing technology. After obtaining positive soybean roots, DNA was extracted and subjected to PCR amplification with specific primers followed by sequencing. Sequencing validation confirmed that *GmHSP26* was successfully edited (substitution and deletion mutations) in the transformed soybean roots (Figure 8A). *GmHSP26* in the roots exhibited a long-fragment deletion. Although the second target site did not result in amino acid changes, the knockout efficiency at target site 1 was approximately 90%, confirming successful knockout of *GmHSP26* and its suitability for subsequent phenotypic analysis. At 14 dpi, the average nodule number per hairy root was 10.18 in CK, compared to 3.52 in the *GmHSP26*-CR line, representing a significant reduction relative to the control. This indicates that *GmHSP26*-CR significantly reduces the nodule number in soybean roots following rhizobial inoculation (Figure 8B,C).

Based on the overexpression and gene knockout results described above, we conclude that *GmHSP26* plays a positive role in regulating soybean nodulation, as its overexpression significantly increases nodule number while its knockdown or knockout markedly reduces it.

## 3. Discussion

Nodulation and nitrogen fixation in legumes represent one of the most efficient biological nitrogen fixation systems in nature and are a core component of the global biological nitrogen cycle. This process is regulated by multiple factors, among which plant growth regulators have become a research hotspot in recent years due to their critical roles in nodulation regulation. Previous studies have shown that gibberellin can induce the expression of *NIN* by binding to cis-acting elements in its promoter, thereby regulating nodulation in *Lotus japonicus* [24]; phenoxyacetic acid activates the expression of *GmGA2ox10*, which inactivates gibberellin and promotes infection thread formation, revealing for the first time the mechanism by which phenoxyacetic acid-type small molecule growth regulators regulate soybean nodulation [25]. TRIA, as an emerging plant growth regulator, plays important roles in regulating plant growth and development, dry matter accumulation, and stress tolerance. Naeem et al. found that TRIA treatment of hyacinth bean significantly increased nitrogenase activity in the nodules, as well as plant biological yield, photosynthetic efficiency, and quality [22]. The present study further revealed that 0.5 μg/mL TRIA treatment significantly increases soybean nodule number and exerts positive effects on the antioxidant system. High concentrations of TRIA produced an effect of reducing the number of nodules, possibly because higher concentrations of TRIA induced an excessive defense response in the plant host, thereby compromising the optimal state of nodulation. PGRs not only influence the nodulation process in legumes but are also closely associated with the levels of reactive oxygen species (ROS) in plants. ROS play a dual role in plant growth and development, with moderate fluctuations serving as important signaling molecules [26]. Exogenous cytokinin increases the activities of POD and CAT (Catalase) in zucchini, indicating its important role in promoting antioxidant enzyme activity [27]. *OsRR26* is involved in mediating ROS accumulation and hormone signaling responses, regulating salt tolerance in rice through the cytokinin signaling pathway [28]. The present study found that TRIA significantly activates SOD and POD activities in soybean roots, while MDA content increased only at 3 dpi, suggesting that TRIA enhances the antioxidant capacity of plants.

ROS also play a critical role throughout the entire lifecycle of nodulation. In the early stages of nodulation, ROS serve as important signal transduction molecules, not only assisting in the formation of infection threads but also activating nodulation-related signaling pathways and initiating the nodulation program [29,30]. During the infection process, ROS are involved in the remodeling of root epidermal cells and interact with auxin-related genes and cyclin-related gene pathways, collectively creating a favorable nodulation microenvironment that promotes nodule development and functional maturation [31]. The RBOHs family is the most extensively studied ROS-related gene family in symbiosis research. For example, both *GmRbohB* and *GmRbohL* influence soybean nodulation and nitrogenase activity by regulating ROS levels [32]. Either excessively low or excessively high ROS levels are detrimental to symbiotic signal transduction and nodule development, and can also reduce nodule nitrogen fixation efficiency, leading to premature nodule senescence and a sharp decline in nitrogen fixation capacity. The transient oxidative peak induced by 0.5 μg/mL TRIA treatment, as observed in this study occurred at the early stage of nodule formation, and this peak was confirmed by NBT staining, further supporting the transient accumulation of ROS (especially superoxide anions) in this process. This controlled oxidative burst is different from the strong oxidative bursts commonly seen during pathogen infection; its intensity is lower and duration is shorter, which may prevent irreversible damage to cells caused by excessive ROS while serving as a signaling molecule to activate downstream nodule-related pathways.

To further elucidate the molecular mechanism by which TRIA regulates nodulation, this study performed transcriptome analysis on soybean root tissues treated with 0.5 μg/mL TRIA at 1, 3, and 5 dpi, as well as control samples. GO enrichment analysis revealed that the DEGs were mainly enriched in terms related to heme binding, tetrapyrrole binding, and oxidation–reduction processes. KEGG enrichment analysis primarily identified pathways such as phenylpropanoid biosynthesis, metabolic pathways, and biosynthesis of secondary metabolites. These results indicate that legume root nodule formation is closely associated with ROS-related pathways and secondary metabolic processes. These results are highly consistent with the previously mentioned early controllable oxidation peaks induced by TRIA. The enrichment of heme/tetrapyrrole-binding proteins (such as peroxidases and catalases) suggests that TRIA not only activates ROS production but also upregulates the antioxidant system to ensure the spatiotemporal controllability of oxidative signals. In addition, the activation of the phenylpropanoid pathway may enhance flavonoid synthesis, promoting rhizobial recognition and infection thread formation, while modulating local phenolic compounds to maintain redox homeostasis. In summary, the transcriptome data molecularly verify the critical mediating role of ROS and related secondary metabolic pathways in the TRIA-promoted soybean nodulation process. Meanwhile, among the 867 DEGs, we screened and observed the expression of nodulation-related genes, and found that there were 17 known nodulation-related genes among them, which responded to TRIA treatment at different time points. Furthermore, WGCNA was performed for all genes and treatments. Based on the expression trends of genes in response to TRIA treatment, a specific module was selected, and the core differentially expressed hub gene, *GmHSP26*, was identified according to gene connectivity within the module.

*GmHSP26* encodes a small heat shock protein belonging to the glutathione S-transferase (GST) family, an important group of oxidation-related proteins that play critical roles in plant growth, development, and stress adaptation. GmHSP26 contains two GST domains and can cooperatively reduce ROS accumulation by activating downstream antioxidant enzyme networks (SOD, POD, CAT) [33,34]. The GST gene family is closely associated with nodulation and nitrogen fixation in legumes. Studies have shown that GST9 is involved in regulating nodule senescence and thus affects nitrogen fixation efficiency by maintaining the reductive homeostasis of the oxidative environment [35,36,37]. In addition, several small heat shock proteins have also been demonstrated to play important regulatory roles in legume nodulation and nitrogen fixation. For example, *GmHSP23.9* regulates soybean nodule number by affecting the expression of genes involved in the NF signaling pathway and the AON signaling pathway [38]; knockout of *GmHSP17.1* inhibits the tricarboxylic acid cycle, thereby hindering nodule formation [39]. These studies further support the notion that *GmHSP26* may coordinately regulate soybean nodulation by integrating oxidative activity regulation with nodulation-related signaling pathways.

The above expression patterns indicate that *GmHSP26* is highly expressed in roots and nodules and is significantly induced after rhizobial infection. Promoter staining further confirms its expression activity during nodule development. This tissue-specific and inducible expression pattern is highly similar to the previously reported nodule-related genes *GmHSP23.9* [38], suggesting that *GmHSP26* may act as a positive regulator involved in early symbiotic signal responses during soybean nodulation. Functional validation results show that overexpression of *GmHSP26* significantly increases nodule number, whereas knockout results in fewer nodules, thereby supporting its positive regulatory role in nodule formation. The involvement of heat shock protein family members in legume nodulation has been reported; for example, small heat shock proteins play a key role in nodule formation [38,39]. The strong induction of *GmHSP26*, a member of the small heat shock protein family, during the early stages of nodule formation may contribute to alleviating localized stress caused by rhizobial infection while stabilizing key proteins in the nodulation signaling pathway, thus promoting infection and nodule development. Together, the expression and functional evidence indicate that *GmHSP26* is an important positive regulator in the soybean nodulation process. Future studies could explore its direct substrates through protein interaction screening to further elucidate its molecular mechanism in symbiotic nitrogen fixation.

Nodulation and nitrogen fixation in legumes are of great significance for energy accumulation, growth and development, and the advancement of green and sustainable agriculture. As a novel plant growth regulator, TRIA can enhance crop yield and disease resistance by influencing plant photosynthesis and oxidative activity. This study demonstrates for the first time that exogenous application of TRIA significantly promotes soybean nodulation. Through comparative transcriptome analysis and molecular functional validation, we preliminarily elucidated the function and mechanism by which *GmHSP26* responds to TRIA-regulated soybean nodulation. These findings provide key insights into the genetic basis and molecular mechanisms underlying the effect of TRIA on nodulation and nitrogen fixation, and offer important theoretical guidance for improving legume nodulation and nitrogen fixation capacity through the application of plant growth regulators, as well as for breeding high-efficiency, high-yield legume crops.

## 4. Materials and Methods

### 4.1. Plant Materials and Growth Conditions

All experiments were performed using the soybean ecotype Fenhei No.1 Seeds were germinated in vermiculite containers (10 cm × 10 cm × 7 cm) under controlled environmental conditions: a 14 h light period at 27 °C ± 0.2 °C and a 10 h dark period at 22 °C ± 0.2 °C, with relative humidity maintained at 50% ± 3%. Upon the full expansion of true leaves, seedlings were transplanted into sterilized vermiculite, inoculated with USDA110 soybean rhizobia (USDA110 is a strain of soybean rhizobia (*Bradyrhizobium japonicum*), and the inoculation volume is 30 mL/plant. For all inoculation steps in this experiment, the rhizobial strain (USDA110) was used and diluted to an OD_600_ of 0.08–0.1), at 12 h after rhizobial inoculation, TRIA was applied via root drench at concentrations of 0, 0.33, 0.5, 1, and 2 μg/mL (30 mL/plant). Nodulation status was evaluated at 14 and 28 dpi.

For seedlings with fully expanded true leaves, rhizobia inoculation was performed. Samples were collected at 0, 1, 3, 6, 9, 12, 24 hpi and at 3, 6, 9, 14, 28 dpi. Additionally, at 14 dpi, various tissues (roots, stems, leaves, and nodules) of seedlings were sampled for gene expression analysis.

Following the inoculation of seedlings at the stage of fully expanded true leaves with rhizobia, uniformly growing plants were selected and subsequently exposed to TRIA at concentrations of 0 or 0.5 μg/mL for treatment durations of 1, 3, and 5 dpi. Root tissues were collected at each time point for transcriptome sequencing, NBT staining analysis, and determination of antioxidant enzyme indicators.

### 4.2. Determination of Antioxidant Enzyme Activity and NBT Staining

Four similarly growing plants per treatment were selected at 1, 3, and 5 dpi after treatment. Root tissues from the same four plants were used to determine antioxidant enzyme activities (SOD, POD) and MDA content, following the protocols of Solarbio Biotechnology kits (BC0175 for SOD, BC0095 for POD, and BC0025 for MDA) (Solarbio Science & Technology Co., Ltd. Beijing, China). For visual detection of superoxide anion in root tips, a 0.1% NBT solution (pH 7.8) (Solarbio Science & Technology Co., Ltd., Beijing, China) was used. Briefly, root tips were immersed in the NBT solution and incubated at 25 °C for 2 h, then rinsed several times with distilled water and observed under a microscope.

### 4.3. cDNA Library Preparation and Illumina Sequencing

All samples were frozen in liquid nitrogen and stored at −80 °C to preserve RNA integrity. Total RNA was extracted from roots treated with 0 or 0.5 μg/mL TRIA at 1, 3, and 5 dpi using RNAiso Plus (Takara Bio Inc., Kyoto, Japan), with three biological replicates per condition. RNA purity, concentration, and integrity were assessed using a Nanodrop2000 (Thermo Fisher Scientific, Waltham, MA, USA). Qualified RNA samples were used to construct cDNA libraries by Guangzhou GENEDENOVO Biotechnology Co., Ltd. (https://www.genedenovo.com/, Guangzhou, China), and sequencing was performed on the NovaSeq X Plus system (Illumina, San Diego, CA, USA).

### 4.4. Transcriptome Data Processing and WGCNA

Raw reads were filtered using fastp (v0.18.0) to remove adapter-containing reads, reads with >10% N, reads consisting entirely of A bases, and reads with >50% of bases having Q ≤ 20. Clean reads were aligned to the *Glycine max* reference genome (Wm82.a4.v1) using HISAT2 (v2.1.0) with default parameters. rRNA-mapped reads were removed using bowtie2 (v2.4.4) against a ribosomal database (no mismatches allowed). Gene expression was quantified using StringTie (v1.3.4) and normalized to FPKM using Salmon (v1.10.3). Differential expression between TRIA (0.5 μg/mL) and control (0 μg/mL) at 1, 3, and 5 dpi was analyzed with DESeq2 (v1.24). Genes with |log_2_ fold change| > 1 and raw *p* < 0.05 were considered differentially expressed. GO enrichment analysis was performed by mapping DEGs to GO terms (http://www.geneontology.org/) using a hypergeometric test, with raw *p* < 0.05 as significance threshold (no multiple testing correction). KEGG enrichment analysis was similarly performed using the KEGG database (https://www.kegg.jp/) with the same hypergeometric test and raw *p* < 0.05. The background gene set for both enrichments was all annotated genes in the reference genome. Three biological replicates were used per condition.

The WGCNA settings are as follows: a co-expression network was constructed using the WGCNA package (v1.47) in R. Genes with a raw read count <1 in more than half of the samples were filtered out. An unsigned network was built using the blockwiseModules function with the following parameters: soft-thresholding power = 8 (scale-free topology criterion), merge similarity = 0.75 (corresponding to mergeCutHeight = 0.25), and minModuleSize = 50. A total of 22 co-expression modules were identified. Module eigengene was defined as the first principal component (PC1) of the read count matrix of all genes within a module, representing the overall expression pattern of that module. Module eigengenes were correlated with sample traits (treatment and time points), and the yellow4 module showing the strongest association with TRIA treatment was selected. Hub genes within this module were identified based on MM > 0.9.

### 4.5. Quantitative Real-Time PCR Analysis

Reverse transcription was performed using the All-In-One RT MasterMix (TransGen Biotech Co., Ltd., Beijing, China) according to the manufacturer’s instructions. qPCR was conducted with 2 × Q3 SYBR qPCR Master Mix (Tolo Biotech Co., Ltd., Shanghai, China) and gene-specific primers. The amplification protocol was: 95 °C for 2 min, followed by 45 cycles of 95 °C for 5 s, 60 °C for 30 s, and 72 °C for 30 s. *CYP2* (*Glyma.12G024700*) was used as the reference gene for normalization [40]. Relative expression levels were calculated using the 2^−ΔΔCT^ method [41]. All qPCR primers are listed in Supplementary Table S3.

### 4.6. Vector Construction and Hairy Root Transformation of Soybean

To construct *GmHSP26* overexpression vectors, the 675 bp coding sequence was amplified from soybean cDNA using 2× Phanta Max Master Mix (Vazyme Biotech Co., Ltd., Nanjing, China). The amplicon was then cloned into the pUBI-GFP vector via *Xba* I and *Kpn* I sites, placing it under the control of the Ljubq1 promoter. Soybean hairy root transformation mediated by *A. rhizogenes* strain K599 was performed as previously described with modifications [42].

For CRISPR-Cas9 mutagenesis, two target sequences (GTGTTTGTTCACAATGAGC and TTCATTGATGATAAGATTG) were designed using Benchling (https://www.benchling.com/, accessed on 18 May 2025.) [43]. These targets were individually inserted into sgRNA expression cassettes, each driven by a U6 promoter, and assembled into the pHSE401-2gR vector. In the functional validation of soybean hairy roots, the control group consists of empty vector-transformed plants for each vector, rather than a single common control.

For the *proGmHSP26-GUS* reporter construct, a 2052 bp genomic fragment upstream of the *GmHSP26* was amplified from genomic DNA and inserted upstream of the *GUS* coding sequence in pCAMBIA1391 using *Hind* III and *BamH* I. Positive transgenic hair roots were screened using GUS staining and phenotypic analysis. The samples were placed in GUS working solution (100 mM Sodium phosphate buffer (pH 7.0), 0.5 mg/mL X-Gluc (dissolved in DMSO), 0.5 mM each of potassium ferricyanide and potassium ferrocyanide, 0.1% (*v*/*v*) Triton X-100) (Coolaber Technology Co., Ltd., Beijing, China) at 37 °C in the dark for 12 h, and then observed after removing the storage solution.

To generate GmHSP26-GFP fusion protein, the *GmHSP26* coding region was amplified and subcloned into the *Hind* III and *Sal* I sites of the pSuper1300-GFP vector. For subcellular localization assays in *N. benthamiana*, the constructed plasmids were introduced into *Agrobacterium tumefaciens* strain GV3101. Positive transformants were then infiltrated into leaf epidermal cells of *N. benthamiana*. The inoculated plants were incubated for 48 h prior to observation. Fluorescence signals were visualized using a Leica confocal laser scanning microscope (Leica Microsystems, Wetzlar, Germany). GFP and mCherry were excited at 488 nm and 587 nm, respectively. All gene-specific primers are listed in Supplementary Table S3.

### 4.7. Statistical Analysis

The resulting data were analyzed with Student’s *t*-test or one-Way ANOVA followed by Tukey’s HSD post hoc test and Dunnett’s test for multiple comparisons with GraphPad Prism 8. The same letter indicates no significant difference between two groups, while different letters indicate a significant difference between two groups, with *p* ≤ 0.05 considered statistically significant. The “ns” indicates no significant difference (*p* > 0.05), * represents *p* ≤ 0.05; ** represents *p* ≤ 0.01; *** represents *p* ≤ 0.001. All data are presented as mean ± standard deviation (Mean ± SD). The nodulation phenotypes under TRIA treatment, related oxidative and physiological indicators, and relevant quantitative data in this experiment were subjected to multiple biological and technical replicates to ensure the accuracy of the obtained results.

## Figures and Tables

**Table 1:** WGCNA.

Module	Gene Number
MM.coral1	885
MM.plum	576
MM.violet	211
MM.darkolivegreen4	251
MM.bisque4	7071
MM.yellow4	316
MM.black	2805
MM.darkseagreen3	199
MM.darkseagreen4	137
MM.darkgrey	319
MM.sienna4	142
MM.darkturquoise	3187
MM.mediumpurple4	676
MM.firebrick3	58
MM.floralwhite	471
MM.coral	190
MM.blue	1887
MM.blue2	1393
MM.salmon4	150
MM.orangered1	892
MM.plum3	1266
MM.grey	24

## Data Availability

The transcriptome sequence data has been deposited in the https://www.cncb.ac.cn/ database with accession number subCRA067943.

## Supplementary Materials

The following supporting information can be downloaded at: https://www.mdpi.com/article/10.3390/plants15101572/s1. Figure S1: Nodule phenotype of soybean at 14 dpi under TRIA treatment. (A) Root nodulation phenotype at 14 days under 0 μg/mL and 0.5 μg/mL TRIA treatment. Scale bar = 10 cm; (B) Statistics of soybean nodule number under gradient concentrations of TRIA treatment at 14 dpi (n = 6–10). Tukey HSD multiple comparisons (α = 0.05) results are marked with letters; different lowercase letters indicate significant differences between treatments (*p* ≤ 0.05), while the same letters indicate no significant difference. (C) Nodule number at 14 dpi under 0 μg/mL and 0.5 μg/mL TRIA treatment. Scale bar = 1 cm; Figure S2. Transcriptome quality analysis. (A) PCA analysis of TRIA and CK treatments at 1, 3, and 5 dpi. (B) Correlation heatmap of the TRIA and CK treatment groups at 1, 3, and 5 dpi; Figure S3. Go enrichment analysis of upregulated DEGs at 1 (A), 3 (C), and 5 (E) dpi; Go enrichment analysis of downregulated DEGs at 1 (B), 3 (D), and 5 (F) dpi; Figure S4. KEGG enrichment analysis of upregulated DEGs at 1 (A), 3 (C), and 5 (E) dpi; KEGG enrichment analysis of downregulated DEGs at 1 (B), 3 (D), and 5 (F) dpi; Figure S5. Correlation analysis of qRT-PCR and RNA-seq data. The dots represent the results of qRT-PCR, and the bars represent the results of RNA-seq data (n = 3); Figure S6. Hierarchical cluster tree showing the 22 modules obtained by weighted gene co-expression network analysis (WGCNA); Figure S7. Analysis of trait-module MM-GS; Figure S8. Colocalization analysis of 35S-GFP and GmHSP26-GFP with 35S-NLS-mCherry (nuclear marker) and 35S-Cyt-mCherry (cytoplasmic marker) in *N. benthamiana* leaf cells (n = 3). Scale bars = 50 µm; Table S1: Transcriptome quality control analysis; Table S2: Yellow4 core gene screening (module-MM > 0.9); Table S3: Primers used in this experiment.

## Abbreviations

The following abbreviations are used in this manuscript:

AON	autoregulation of nodulation
CAT	Catalase
DEGs	differentially expressed genes
dpi	days post inoculation
GO	Gene Ontology
GST	glutathione S-transferase
hpi	hours post inoculation
KEGG	Kyoto Encyclopedia of Genes and Genomes
MDA	Malondialdehyde
MM	module membership
NBT	Nitroblue Tetrazolium
PGRs	plant growth regulators
POD	Peroxidase
ROS	reactive oxygen species
SNF	symbiotic nitrogen fixation
SOD	Superoxide Dismutase
TRIA	Triacontanol
WGCNA	weighted gene co-expression network analysis
