# Supplementary material for: Triacontanol Boosts Soybean Nodulation via GmHSP26-Mediated Antioxidant Enhancement
Source: Plants (Basel). 2026 May 21;15(10):1572. doi: 10.3390/plants15101572 (PMC13210726; doi:10.3390/plants15101572)
Supplement: Supplementary file 1 [file plants-15-01572-s001.zip › Supplementary Figure.pdf]

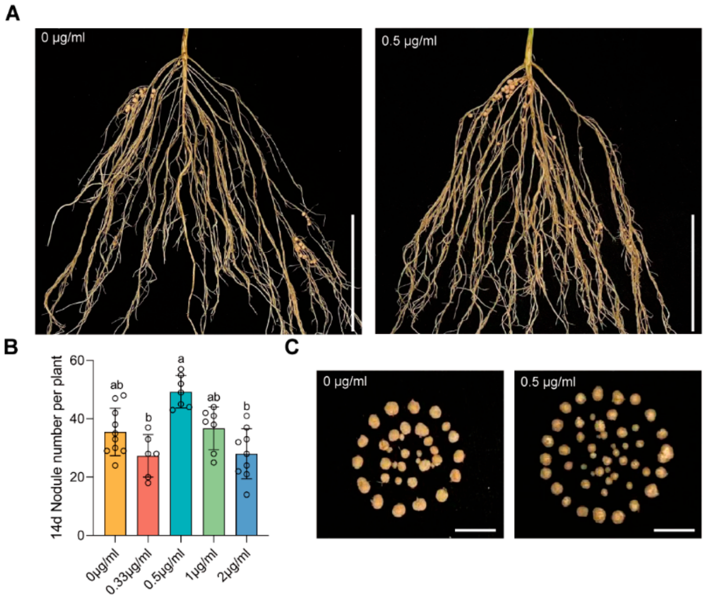

Figure S1. Nodule phenotype of soybean at 14 dpi under TRIA treatment. (A) Root nodulation phenotype at 14 days under 0 μg/mL and 0.5 μg/mL TRIA treatment. Scale bar = 10 cm; (B) Statistics of soybean nodule number under gradient concentrations of TRIA treatment at 14 dpi (n = 6-10). Tukey HSD multiple comparisons (α = 0.05) results are marked with letters; different lowercase letters indicate significant differences between treatments ($P \leq 0.05$), while the same letters indicate no significant difference. (C) Nodule number at 14 dpi under 0 μg/mL and 0.5 μg/mL TRIA treatment. Scale bar = 1 cm.

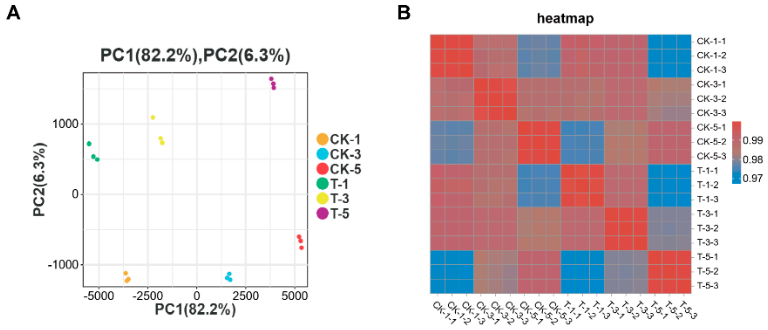

Figure S2. Transcriptome quality analysis. (A) PCA analysis of TRIA and CK treatments at 1, 3, and 5 dpi. (B) Correlation heatmap of the TRIA and CK treatment groups at 1, 3, and 5 dpi.

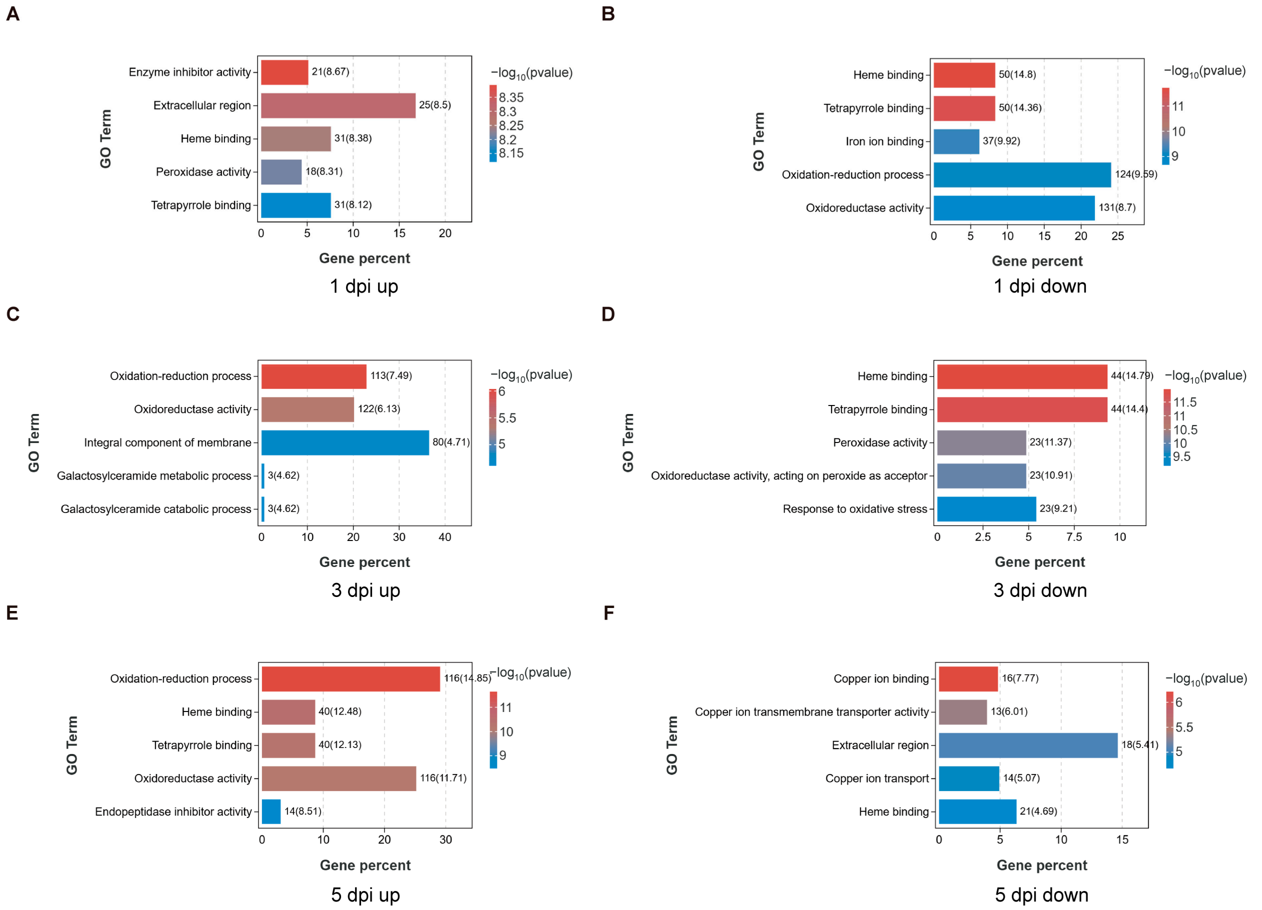

Figure S3. Go enrichment analysis of upregulated DEGs at 1 (A), 3 (C), and 5 (E) dpi; Go enrichment analysis of downregulated DEGs at 1 (B), 3 (D), and 5 (F) dpi.

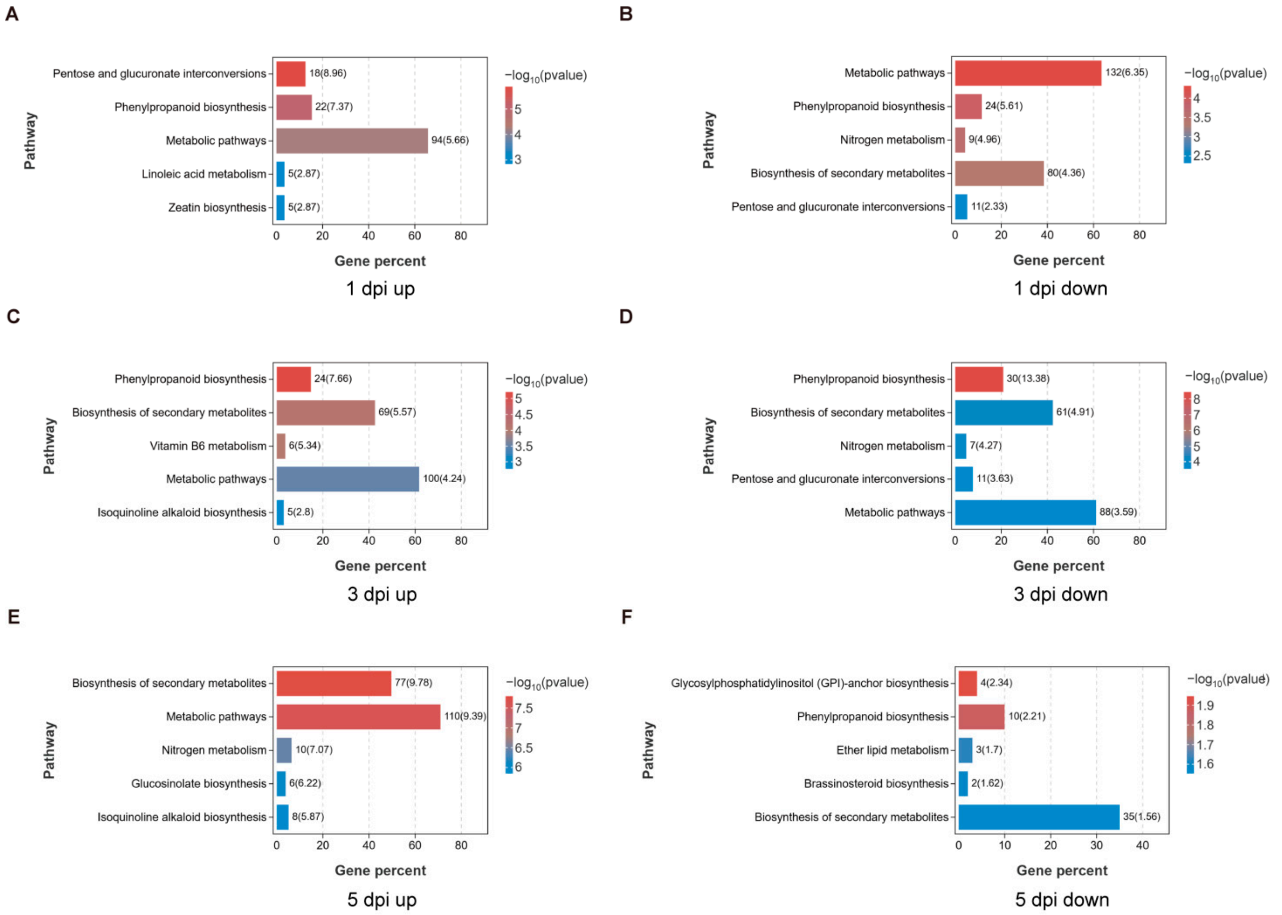

Figure S4. KEGG enrichment analysis of upregulated DEGs at 1 (A), 3 (C), and 5 (E) dpi; KEGG enrichment analysis of downregulated DEGs at 1 (B), 3 (D), and 5 (F) dpi.

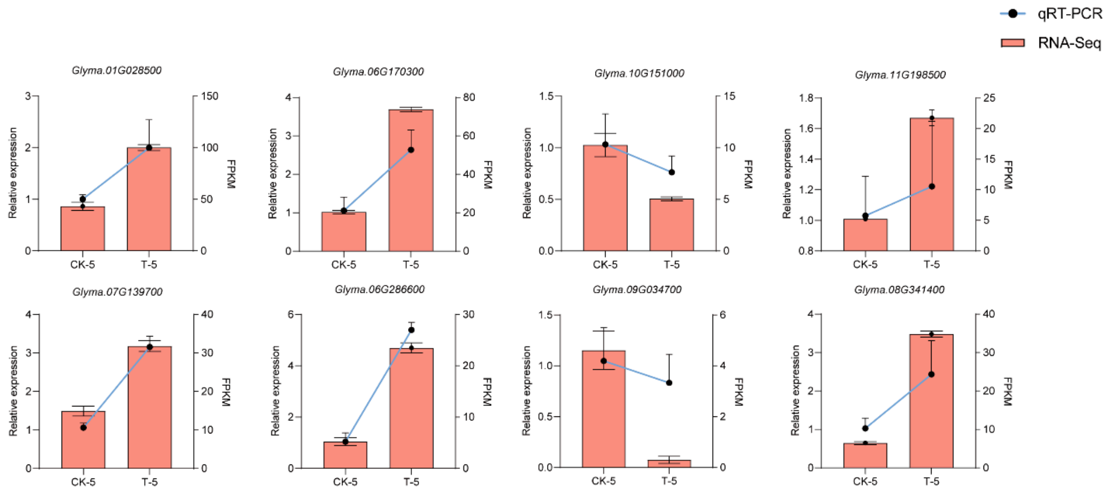

Figure S5. Correlation analysis of qRT-PCR and RNA-seq data. The dots represent the results of qRT-PCR, and the bars represent the results of RNA-seq data (n = 3).

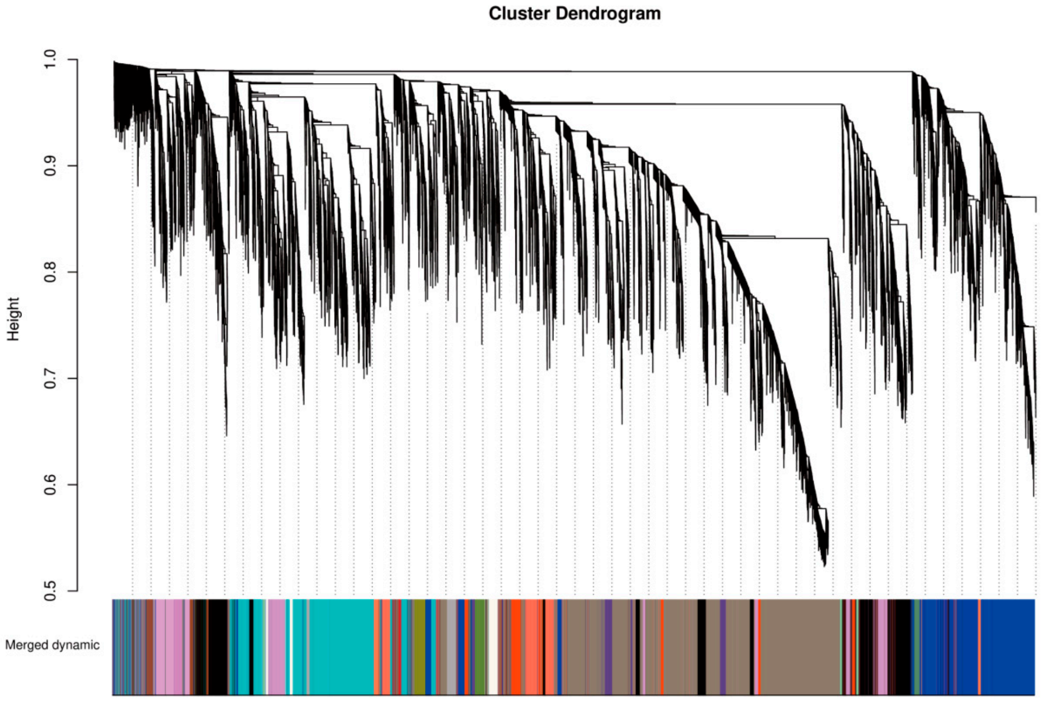

Figure S6. Hierarchical cluster tree showing the 22 modules obtained by weighted gene co-expression network analysis (WGCNA).

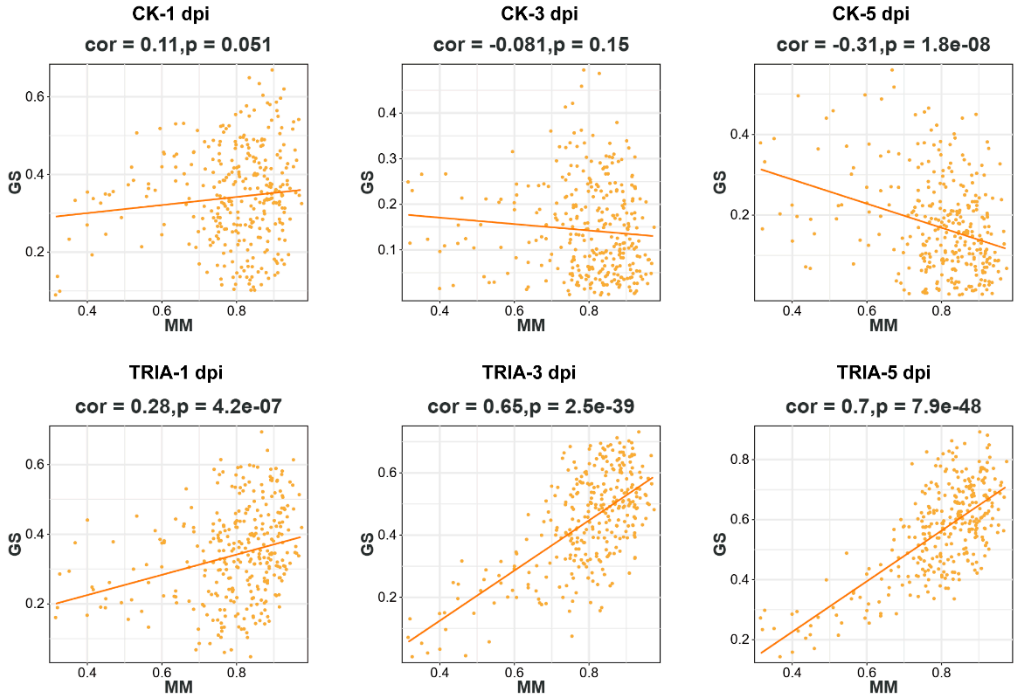

Figure S7. Analysis of trait-module MM-GS.

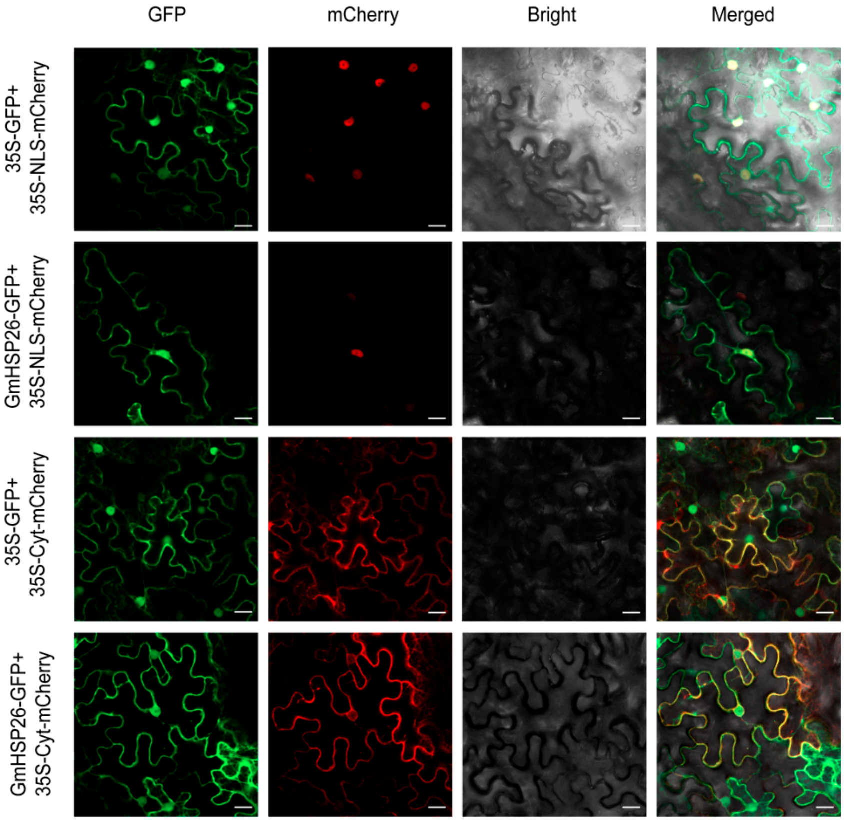

Figure S8. Colocalization analysis of 35S-GFP and GmHSP26-GFP with 35S-NLS-mCherry (nuclear marker) and 35S-Cyt-mCherry (cytoplasmic marker) in *N. benthamiana* leaf cells (n = 3). Scale bars = 50 μm.
